# Age-related changes in regiospecific expression of Lipolysis Stimulated Receptor (LSR) in mice brain

**DOI:** 10.1371/journal.pone.0218812

**Published:** 2019-06-24

**Authors:** Aseel El Hajj, Frances T. Yen, Thierry Oster, Catherine Malaplate, Lynn Pauron, Catherine Corbier, Marie-Claire Lanhers, Thomas Claudepierre

**Affiliations:** Qualivie, UR AFPA laboratory, ENSAIA, University of Lorraine, Vandoeuvre-les-Nancy, Lorraine, France; Universite de Technologie de Compiegne, FRANCE

## Abstract

The regulation of cholesterol, an essential brain lipid, ensures proper neuronal development and function, as demonstrated by links between perturbations of cholesterol metabolism and neurodegenerative diseases, including Alzheimer’s disease. The central nervous system (CNS) acquires cholesterol via *de novo* synthesis, where glial cells provide cholesterol to neurons. Both lipoproteins and lipoprotein receptors are key elements in this intercellular transport, where the latter recognize, bind and endocytose cholesterol containing glia-produced lipoproteins. CNS lipoprotein receptors are like those in the periphery, among which include the ApoB, E binding lipolysis stimulated lipoprotein receptor (LSR). LSR is a multimeric protein complex that has multiple isoforms including α and α’, which are seen as a doublet at 68 kDa, and β at 56 kDa. While complete inactivation of murine *lsr* gene is embryonic lethal, studies on *lsr* +/- mice revealed altered brain cholesterol distribution and cognitive functions. In the present study, LSR profiling in different CNS regions revealed regiospecific expression of LSR at both RNA and protein levels. At the RNA level, the hippocampus, hypothalamus, cerebellum, and olfactory bulb, all showed high levels of total *lsr* compared to whole brain tissues, whereas at the protein level, only the hypothalamus, olfactory bulb, and retina showed the highest levels of total LSR. Interestingly, major regional changes in LSR expression were observed in aged mice which suggests changes in cholesterol homeostasis in specific structures in the aging brain. Immunocytostaining of primary cultures of mature murine neurons and glial cells isolated from different CNS regions showed that LSR is expressed in both neurons and glial cells. However, *lsr* RNA expression in the cerebellum was predominantly higher in glial cells, which was confirmed by the immunocytostaining profile of cerebellar neurons and glia. Based on this observation, we would propose that LSR in glial cells may play a key role in glia-neuron cross talk, particularly in the feedback control of cholesterol synthesis to avoid cholesterol overload in neurons and to maintain proper functioning of the brain throughout life.

## Introduction

Cholesterol is essential for neuronal physiology; a tight regulation of cholesterol homeostasis in the central nervous system (CNS) is essential for proper neuronal development and function. Cholesterol is not only an important structural component for cellular membranes and myelin, it is also required for synapse and dendrite formation [1,2], and axonal guidance [3]. It ensures functional synaptogenesis and is vital for: synaptic vesicles transmission along axonal microtubules *via* cholesterol-kinesin interactions, exocytotic complex organization in active presynaptic membranes of lipid rafts, neurotransmitter receptors clustering in postsynaptic membranes, extra-synaptic receptors pool recruitment, and pre- and post-synaptic cell-cell adhesion [4]. Cholesterol is delivered to tissues *via* lipoprotein particles. However, access to the brain of cholesterol and other lipids from the peripheral circulation is limited due to the blood–brain barrier (BBB), which serves as a selective low-permeable multicellular barrier [5]. Cholesterol is therefore supplied by *de novo* synthesis in the brain, which relies on its own network for synthesizing, internalizing and metabolizing these lipids to provide the necessary components for neuronal cell membrane function [5,6]. Glial cells play a central role towards providing neurons with lipids, particularly cholesterol in the form of lipoproteins. Lipoproteins in the cerebrospinal fluid (CSF) are very different from those of the periphery, and have been characterized as high-density lipoprotein-like (HDL-like) particles containing primarily apolipoprotein (Apo)E and ApoJ [7]. These HDL-like lipoproteins are needed to export cholesterol from astrocytes to neurons, where they bind via ApoE to lipoprotein receptors and are internalized through receptor-mediated endocytosis [4]. A series of lipoprotein receptors expressed in neurons have been identified including the low density lipoprotein receptor (LDL-R) [8], low density lipoprotein receptor-related protein 1 (LRP-1) [8], and lipolysis stimulated lipoprotein receptor (LSR) [9]. LSR is the most recently discovered receptor found to be expressed in the CNS [9]. It is a multimeric protein complex that undergoes conformational changes in the presence of free fatty acids, thereby revealing a binding site that recognizes ApoB or ApoE [10]. There are three different isoforms of LSR that have been clearly identified, LSR α and α’ that form a doublet at 68 kDa, and β at 56 kDa [11]. LSR α is the complete protein sequence of LSR, which contains a clathrin binding site and a di-leucine lysosomal targeting signal at the N-terminal, a hydrophobic transmembrane domain, a cysteine-rich region and a group of alternatively negatively and positively charged amino acids at the C-terminal which serve as the lipoprotein binding site [11]. While LSR α’ has a similar protein sequence to LSR α, the di-leucine lysosomal signal is deleted, but it can still act as a transmembrane protein. However, the LSR β protein sequence does not contain either the transmembrane domain or the dileucine routing motif responsible for internalization and endocytosis; despite this, LSR β is still able to bind lipoproteins in the presence of fatty acids [11]. LSR is plays an important role in mediating hepatic clearance of triglyceride-rich ApoB, E-containing lipoproteins during the post-prandial phase [12]. *In vivo* studies have shown that this receptor is necessary for maintaining normal peripheral circulation levels of cholesterol and triglycerides, and in contributing to the regulation of lipid distribution amongst the peripheral tissues [12,13]. Hepatic LSR’s role in clearance of lipoproteins was further confirmed by the observation of increased plasma levels of cholesterol and triglycerides following shRNA-mediated knockdown of hepatic LSR expression in mice [13]. Complete inactivation of *lsr* is associated with *in utero* lethality at the embryonic stage, most likely due to brain-localized hemorrhages and BBB leakage [14,15]. Complete inactivation of *lsr* is embryonic lethal, however, *in vivo* studies conducted on young and aged *lsr* +/- mice suggest that reduced LSR may be associated with cognitive disturbances related to reactivity to novel environments in aged *lsr* +/- mice [9]. A significant decrease of lipid droplets, which are lipid-rich cellular organelles that regulate the storage and hydrolysis of neutral lipids, including cholesterol [16], was observed in Purkinje cells of the cerebellum (CB) together with an accumulation of filipin-labeled cholesterol in neuronal membranes of the hippocampus (HIP) in aged *lsr* +/- mice [9]. Histochemical studies show a neuron-specific strong expression of LSR in HIP, Purkinje cells, at ependymal cells surface between brain parenchyma and CSF, and in the capillary-rich choroid plexus region [9]. Interestingly, Daneman’s laboratory identified the presence of *lsr* gene transcript in endothelial cells (ECs) of the BBB [17] and showed that LSR is a component of paracellular junctions highly enriched in the BBB ECs, but not in ECs in peripheral tissues outside the CNS [15]. They demonstrated that the BBB doesn’t seal during embryogenesis in *lsr* knockout mice. Another study reported the high expression of LSR in tricellular junctions, not only in the BBB, but also in retinal ECs that form the inner blood retinal barrier (BRB) [18]. This indicates that LSR plays a critical role in maintaining the BBB integrity and suggests a potential role of LSR in the transport of lipoproteins between the brain parenchyma and the CSF. Altogether, the evidence point towards a critical role for LSR in cholesterol trafficking in the CNS during the lifespan. Our objective was to establish a detailed profile of LSR RNA and protein expression in the brain of young and old mice, both on whole brain tissue and in specific CNS areas including the hypothalamus (HT), hippocampus (HIP), olfactory bulb (OB), retina (RET), cortex (CX), and cerebellum (CB). We also compared LSR expression between primary cultures of glial and neuronal cells from different CNS regions. This study revealed differential expression of LSR isoforms in different regions, which would help us, in the future, understand the possible role of LSR in the cholesterol crosstalk between glial cells and neurons.

## Materials and methods

### Animals

Three month and eighteen-month-old male and female C57Bl/6JRj mice (Janvier Breeding, Le Genest Saint Isle, France) were used for the study (n = 3 for each group). For primary cell cultures, newborn C57Bl/6JRj mice aged 5–7 days were sacrificed (n = 7–10). The C57Bl/6JRj mice were housed in certified animal facilities (N° B54-547-24) on a 12-h light/dark cycle with a mean temperature of 21–22°C and relative humidity of 50 ± 20% and provided rodent chow diet (16.4% protein, 4% fat, ref 2016, Envigo Teklad, Gannat, France) and water *ad libitum*. Animal care followed French State Council guidelines for the use and handling of animals: all tissues used in the study were collected after sacrifice of animals using isoflurane anesthesia followed by decapitation in order to preserve the nervous structures for further histological analysis. In the frame of a larger behavioral study, this protocol has been approved by the Local Ethical Committee (CELMEA, agreement number APAFIS#12079-201711081110404v2). All personals in contact with animal has been trained and detain an Authorization for Animal Experimentation from the French Authorities.

### Immunoblots

Tissues were collected, and different brain regions were isolated: HT, HIP, OB, RET, CX, and CB. Whole cell extracts were prepared using RIPA lysis Buffer (10x RIPA buffer, ref 20–188 Millipore, Darmstadt, Germany) supplemented with 10 mM sodium orthovanadate (ref S6508, Sigma Aldrich, Saint-Quentin Fallavier, France), 10 mM phenylmethylsulfonyl fluoride (ref P7626, Sigma Aldrich), and protease inhibitors (ref 11 836 145 001, Roche, Mannheim, Germany). The concentration of the isolated proteins was determined using Pierce BCA Protein Assay Reagent (ref 3225 Thermofisher Scientific, Villebon-sur-Yvette, France). Twenty micrograms of the protein were separated on a 10% SDS-PAGE and electrophoretically transferred to nitrocellulose membranes (ref 10600003, Sigma Aldrich). Membranes where then incubated with primary polyclonal antibodies against the LSR protein; LSR Sigma (1:500 rabbit, ref HPA007270-100UL, Sigma Aldrich), and against the control protein β-Tubulin (β-TUB) using a mouse monoclonal antibody (1:1000, mouse, ref T5201, Sigma Aldrich). LSR was detected with HRP-conjugated sheep anti-rabbit IgG antibody (ref 7074, 1:2000 dilution, Cell signaling technologies, Leiden, Netherlands), whereas β-TUB was detected with HRP-conjugated sheep anti-mouse IgG antibody (ref 7076, 1:2000 dilution, Cell signaling technologies) and visualized with the Luminata Crescendo Western HRP substrate (ref WBLUR0500, Millipore, Molsheim, France), according to manufacturer’s instructions.

### RNA extraction and RT-qPCR

Freshly collected tissues were conserved in RNAlater (ref 76104, Qiagen, Les Ulis, France) as per manufacturer’s instructions and stored at -80°C until use. Different regions of the brain were isolated separately including HT, HIP, OB, RET, CX, and CB. Total RNA was extracted using TRI reagent (ref T9424, Sigma Aldrich), according to the manufacturer’s instructions. RNA quantity and purity were estimated by a Nanodrop ND-1000 spectrophotometer (Thermo Scientific; Villebon-sur-Yvette, France), and the samples with a 260/280 nm ratio ≥ 1.7 were used for subsequent analyses. RNA quality was verified by bleach agarose gel electrophoresis [19]. RNA samples showing intact 28S and 18S ribosomal subunits were considered suitable for further cDNA synthesis. Reverse transcription was performed using 1 μg of RNA in a final volume of 20 mL including 0.5 mL of random primers (3 mg/mL; Invitrogen, Carlsbad, CA, USA), 1 μL of 10 mM dNTP mix, in RNase-free water (ref 10977049, Invitrogen, Cergy Pontoise, France). After denaturation of RNA samples at 65°C for 5 min, 4 μL of buffer (5x), 2 μL of 0.1 mM DTT, 1 μL of Superscript II reverse transcriptase (ref 18064022, Invitrogen), and 1 μL of RNase OUT (ref 10777019, Invitrogen) were added. Samples were homogenized and were transcribed in an Applied Biosystems 2720 thermal cycler according to the following conditions: 25°C for 10 min, 42°C for 50 min, and 70°C for 15 min. The cDNA from individual animals was used as a template for the PCR array using the Applied Biosystem kit (ref A25742, Thermofischer Scientific) with the following final concentrations in a 25 μL final volume: 1 × Master Mix, 100 nM forward and reverse primers, 0.4 ng/μL cDNA. The mix was placed in a 7500 Fast Real-Time PCR system (Applied Biosystems; Foster City, CA, USA). The thermal cycling conditions were: initial 5 min denaturation at 95°C, followed by 42 cycles of 15 s at 95°C, 1 min at 60°C, and a final dissociation step. The primer specificity was determined based on the presence of a single peak in the melting curve. We followed four target mRNA sequences: total *lsr*, *lsr* α, *lsr* α’, and *lsr* β, whose expression levels were compared to those of three reference sequences: hypoxanthine guanine phosphoribosyl transferase (*Hprt*) [12], phosphoglycerate kinase 1 (*Pgk1*) [20], and transferrin receptor protein 1 (*Tfrc1*) [20] (S1 Table). *Lsr* primer sequences were selected using the Primer-BLAST Genbank based on *lsr* gene sequence (NM_017405). Quantitation was performed by the 2^-ΔΔCt^ method [21]. The obtained results were tested for statistical significance (p<0.05) using the Relative Expression Software Tool 2009 (REST Version 2.0.13). Fold changes of mRNA samples of young animals were compared to whole brain levels, and those of old 18-month-old animals were compared to that of 3-month-old ones.

### Mixed cell culture

Different brain structures (HT, HIP, OB, RET, CX, and CB) were collected from mice (n = 7–10 mice) of 5–7 days of age immediately after sacrifice. Tissues were collected in D-PBS and were cut into small pieces of 1 mm^3^. Afterwards, tissues were digested using papain digestion solution containing 20 U/mL papain (ref LS003126, Worthington Biochemical Corporation, Lakewood, NJ, USA) and 200 U/mL DNase (ref D4527, Sigma Aldrich), for 1 hour at 37°C with constant rotation. This was followed by mechanical dissociation of the pellet for 3 times using a 0.02% (w/v) BSA solution (ref A4161, Sigma Aldrich) containing 333 U/mL DNase (ref D4527, Sigma Aldrich). This was followed by cell counting using a hemocytometer and centrifugation at 1000 rpm for 10 minutes. Cells were resuspended in a glia culture medium at 1000 cells/μL and plated at 200,000 cells/glass slide. Culture medium was DMEM medium (ref 41965–039, Gibco Life technologies,) supplemented with 10% fetal bovine serum (ref 16000–044, FBS-Invitrogen/Gibco), 2 mM glutamine (Sigma, G7513), 1 mM sodium pyruvate (ref 11360–039, Invitrogen/Gibco), 100 U/mL penicillin and 100 μg/mL streptomycin (Pen/Strep solution, ref 151 40–122, Invitrogen/Gibco). Cells were incubated at 37°C with 5% CO_2_ for 8 days and medium was changed every 3 days.

### Immunoisolation and culture of CNS neurons from postnatal mice

Neurons were isolated from HT, HIP, RET, OB and CB of freshly sacrified postnatal C57Bl/6J mice (n = 7–10) of 5–7 days of age with an immunopanning technique, according to a previously published protocol [22]. Mice were sacrificed by decapitation according to institutional guidelines. Different tissues were collected in D-PBS and were cut into small pieces of 1 mm^3^. Afterwards, tissues were digested using papain digestion solution, which contains 33 U/mL papain (ref LS003126, Worthington Biochemical Corporation, Lakewood, NJ, USA) and 200 U/mL DNase (ref D4527, Sigma Aldrich), for 1 hour at 37°C with constant rotation. The tissues were then sequentially triturated in a 0.02% bovine serum-albumin (BSA, ref A4161, Sigma Aldrich) containing 333 U/mL DNase (ref D4527, Sigma Aldrich). Cells were filtered using a wet nylon mesh (Nitex 20 *μ*m, Tetko/Sefar Filtration, Rüschlikon, Switzerland) with 0.02% BSA, then spun down (1000 *rpm* for 10 min) and resuspended with 15 mL 0.02% BSA (ref A4161, Sigma Aldrich). For immunopanning, one (HT, HIP, CB, OB, and CX) or two (Retinal ganglion cells-RGCs) subtraction plates (150 mm diameter Petri-dishes; Falcon; BD Biosciences/VWR, Fontenay sous Bois, France) and one selection plate (100 mm diameter Petri-dish) were incubated for *>*12 h at 4°C with 10 *μ*g/mL secondary antibody in 50 mM Tris-HCl (pH 9.5). For subtraction: goat anti-rabbit IgG (ref 111-005-003, Jackson Immunoresearch Laboratories, Marseille, France); for selection: HT, HIP, CB, OB, CX, goat anti-rat IgG (ref 112-005-003, Dianova, West Grove, PA, USA); for RGCs, goat anti-mouse IgM (ref 111-005-020, Jackson Immunoresearch Laboratories). After washing for three times with PBS, selection plates were covered with 0.2% BSA (ref A4161, Sigma Aldrich) and incubated for at least 2 h at room temperature (HT, HIP, OB, CX, CB) with 0.2 μg/ mL of rat anti-L1CAM IgG (ref MAB5275, Millipore, Darmstadt, Germany) or (RET) 0.2 μg/mL of mouse IgM anti-Thy1.2 (ref MCA01, Serotec, Cergy Saint-Christophe, France) and then washed with D-PBS. The filtered cell suspension was incubated on subtraction plates for 40 min. The supernatant was filtered and incubated on the selection plate (45–60 min). Non-adherent cells were removed by thorough washing, and bound cells were released using 30% (w/v) fetal bovine serum (ref 16000–044, Gibco/Invitrogen, Cergy Pontoise, France). After centrifugation, cell pellets were resuspended in a fully saturated culture medium (FSM) at 1000 cells/μL and plated at 2.10^4^ cells/well, in 12-well plates, containing coverslips (8 mm diameter) previously coated with poly-orthinine (ref P0421, Sigma), and kept in a humidified chamber at 37°C containing 5% CO_2_ for 8 days *in vitro* (D-VIII). To obtain FSM, neurobasal medium (ref 21103049, Gibco/Invitrogen) was supplemented with (all from Sigma, except where indicated) 1 mM pyruvate (ref 11360–039, Invitrogen/Gibco), 2 mM glutamine (ref G7513), 60 μg/mL *N*-acetyl-l-cysteine (ref A9165), 16 μg/mL putrescine (ref P5780), 40 ng/mL sodium selenite (ref S5261), 100 μg/mL BSA (ref A4161), 100 μg/mL streptomycin and 100 U/mL penicillin (ref 15140–122, Invitrogen/Gibco), 40 ng/mL triiodothyronine (ref T6397), 100 μg/mL holotransferrin (ref T4132), 10 μM forskolin (ref F6886), 5 μg/mL insulin (ref I6634), 62 ng/mL progesterone (ref P8783) and 1:50 B27 (ref 17504–036, Gibco/Invitrogen). This medium is referred to as minimally supplemented medium (MSM). MSM was further supplemented with 25 ng/mL brain-derived neurotrophic factor (BDNF; ref 450–02, PeproTech, London, UK), and 10 ng/mL ciliary neurotrophic factor (CNTF; ref 450–50, PeproTech) necessary for neuronal survival. This medium is referred to as fully supplemented medium (FSM).

### Immunocytochemistry

After fixation with 2% paraformaldehyde (PAF; ref 158217, Sigma) followed by 4% PAF for 20 min each, cells were permeabilized and blocked using a 0.2% Triton (ref T8787, Sigma Aldrich) and 30% Cas-Block (ref 008120, Invitrogen) solution prepared in 1X D-PBS (ref 141900094, Gibco) for about 1 hour at room temperature. This was followed by incubating cells for 2 h at room temperature, with rabbit anti LSR X-25 (1:100, ref sc-133765, Santa-Cruz, CA, USA) and either mouse anti-GFAP (1:500, ref MAB360, Chemicon, CA, USA), mouse anti-AQP4 (1:500, ref sc-32739, Santa-Cruz), or mouse β-tubulin III (β-TUB) (ref 201202, Biolegend, San Diego, USA) prepared in the permeabilization and blocking solution mentioned above. This was followed by incubating cells with Alexa 488-anti mouse (1:500, ref A11001) and Alexa594-anti rabbit (1:500, ref A21428) conjugated secondary antibodies (Molecular Probes, Invitrogen, Cergy Pontoise, France) for 1 h at room temperature. After, cell nuclei were labelled by incubation with DAPI (1:1000, ref D9542, Sigma) for 10 min at room temperature. Finally, the slides were mounted on glass slides using Fluoromount-G (ref 1798425, Electron Microscopy Sciences, Hatfield, PA, USA), and left at room temperature overnight, protected from light. Slides were then examined on a confocal microscope Fluoview FV10i (Olympus, Center Valley, PA, USA).

### Statistical analysis

For immunoblots: the area of bands was calculated using Image J, then the ratio LSR/β-TUB was calculated for each lane, followed by calculation of the mean and standard error. Statistical significance was calculated using t-test ± SEM.

For RT-qPCRs, statistical data in the boxplot were obtained using REST software tool, where (+) represents the mean value, the middle line represents the median, the lower (Q1) and upper (Q3) lines in the bar represent the 25% and 75% quartile, respectively. While the upper and lower lines represent the observations outside the 9–91 percentile range, data falling outside of Q1 and Q3 range are plotted as outliers of the data.

## Results

### *Lsr* RNA profiling in mice brain

#### Variation of *lsr* mRNA levels in different CNS areas

**Fig 1:**
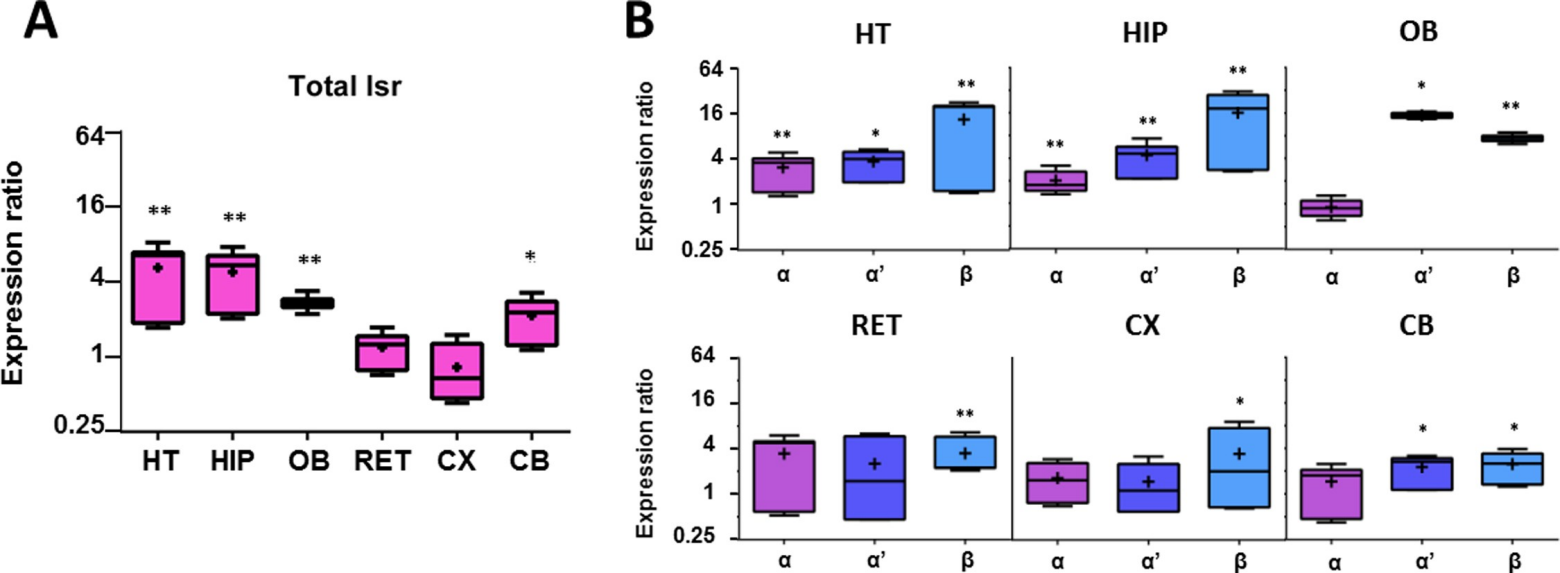
Boxplot of RT-qPCR data of *lsr* mRNA expression in different brain regions of 3-month-old male C57Bl/6JRj mice (n = 3) with respect to whole brain homogenates (n = 3). (A) Box plot of total *lsr* expression in various regions including hypothalamus (HT), hippocampus (HIP), olfactory bulb (OB), retina (RET), cortex (CX), and cerebellum (CB). (B) Expression ratio of different *lsr* isoforms α, α’, and β. Statistical significance is represented as: * p ≤ 0.05, ** p ≤ 0.01, *** p ≤ 0.001.

Expression levels of *lsr* mRNA were estimated after performing RT-qPCR on total RNA fractions extracted from different brain regions. In young 3-month-old males, we observed a more than 4-fold increase in total *lsr* expression in both the HT (*p* = 0.007) and HIP (*p* = 0.007), while in the OB (*p* = 0.007) and CB (*p* = 0.03) there was a 2–2.7 fold increase relative to that measured in whole brain tissues of male mice of the same age (Fig 1A). When comparing different splice variants of *lsr*, *lsr* β mRNA expression was significantly higher than that of the whole brain in all of the above-mentioned regions (Fig 1B). There was more than 7-fold expression in the HT (*p* = 0.007), HIP (*p* = 0.003), OB (*p* = 0.007), and CX (*p* = 0.017), as compared to a 2–3 fold increase in RET (*p* = 0.01), and CB (*p* = 0.026). Concerning *lsr α*, there was a 2-3-fold increase in both the HT (*p =* 0.006), and HIP (*p* = 0.006). Finally, there was a 2-fold increase of *lsr α’* in CB (*p* = 0.013), a 3.5–4.5-fold increase in both the HT (*p* = 0.013) and HIP (*p* = 0.009), and a 15-fold increase in the OB (*p* = 0.013).

#### Age-related changes in *lsr* expression

**Fig 2:**
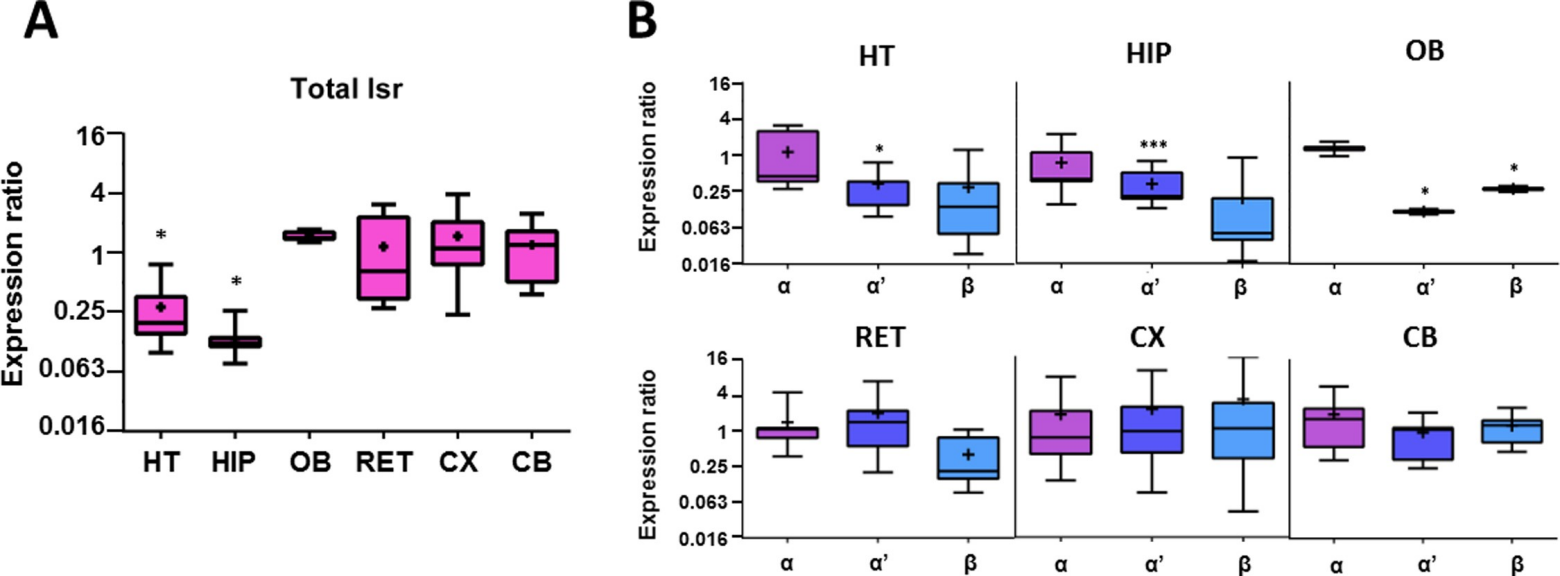
Boxplot of RT-qPCR data of *lsr* mRNA expression in different brain regions of 18-month-old (n = 3) with respect to 3-month-old male C57Bl/6JRj mice (n = 3). (A) Boxplot of total *lsr* expression in various regions including HT, HIP, OB, RET, CX, and CB. (B) Expression ratio of different *lsr* isoforms α, α’, and β. Statistical significance is represented as: * p ≤ 0.05, ** p ≤ 0.01, *** p ≤ 0.001.

To determine if *lsr* expression was modified with age, RT-qPCR analysis was also performed on brain regions from older 18-month old mice, and results were expressed compared to 3-month old males (Fig 2). There was a significant decrease in total *lsr* mRNA expression in both HT (*p* = 0.032) and HIP (*p* = 0.039) where levels were 0.1–0.2-fold as compared to those of 3-month-old mice (Fig 2A), while *lsr* RNA expression remained relatively unchanged in the other structures. When considering the different variants (Fig 2B), *lsr* α’ mRNA expression was significantly reduced in the HT (0.269-fold, *p* = 0.032) and HIP (0.27-fold, *p* = 0.0001). Similarly, *lsr* β showed a tendency to decrease with age in both HT (0.073-fold, *p* = 0.14) and HIP (0.138-fold, *p* = 0.094), but this was not statistically significant (Fig 2B). Although total *lsr* mRNA levels in the OB tended to increase in the older mice (1.469-fold, *p* = 0.07, Fig 2A) and *lsr* α levels were slightly increased (1.251-fold, *p* = 0.288), *lsr* α’ (0.115-fold, *p* = 0.03), and *lsr* β (0.273-fold, *p* = 0.03) levels were significantly decreased (Fig 2B).

RT-qPCR analysis of total *lsr* mRNA levels from brain regions of young female mice revealed no statistically significant differences as compared to those of male mice in RET, CB or HIP. However, total *lsr* mRNA was 4-fold higher (*p* = 0.03) in the CX region, and 4.5-fold lower (*p* = 0.03) in HT region of young female as compared to young male mice, while total *lsr* mRNA was 4.5-fold lower in females compared to young male mice (S1 Fig). Older 18-month old female mice showed no significant variation in total *lsr* expression when compared to 18-month old male mice except in HIP and HT, where there was a 4-fold (*p* = 0.01) and 2.47-fold (*p* = 0.03) increase, respectively (S2 Fig).

### LSR differential expression in different brain regions and the retina

**Fig 3:**
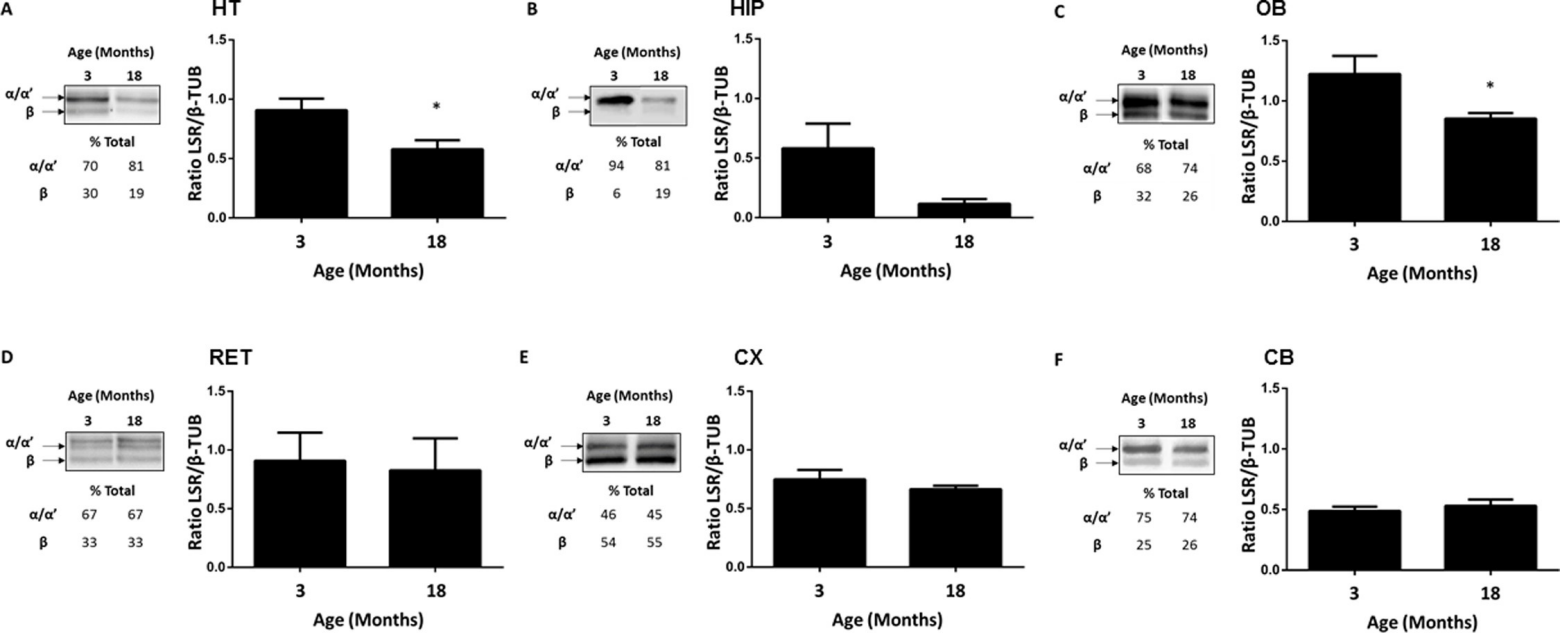
Age dependent changes in LSR protein levels in C57Bl/6JRj male mice. A, B, C, D, E, and F correspond to results obtained for hypothalamus (HT), hippocampus (HIP), olfactory bulb (OB), retina (RET), cortex (CX), and cerebellum (CB), respectively. In each panel there are immunoblots to detect LSR in the different brain structures, calculated percentages of α/α’ and β compared to total LSR, and expression ratio of total LSR with respect to β-TUB from young 3-month old (n = 3), and old 18-month old (n = 3). Statistical significance is represented as: * p ≤ 0.05, ** p ≤ 0.01, *** p ≤ 0.001.

In order to assess LSR protein levels, immunoblots using anti-LSR antibody (LSR Sigma) to detect LSR were performed on protein extracts from different regions of the brains of 18-month old mice, including the HT, HIP, OB, RET, CX, and CB. Interestingly, comparison of LSR protein levels revealed differences in those of LSR subunits α/α’ (68 kDa), and β (56 kDa) in the various CNS regions. Anti-LSR Sigma antibodies (S4 Fig) detected the major two bands corresponding to the α/α’ isoforms, seen as a doublet at 68 kDa, while the β isoform was identified as the lower band migrating at 56 kDa. Interestingly, the different isoforms of LSR (α, α', and β) were not equally expressed throughout the CNS. To examine this, the differences in the relative amounts of the three LSR isoforms (Fig 3, immunoblots and table) or total LSR protein normalized to β-TUB in brain regions from old versus young male mice were compared (Fig 3, bar graphs). For example, the LSR β-chain represents only 10% of total LSR in the HIP (Fig 3B), and more than 50% in the CX (Fig 3E).

When normalized to β-TUB, by calculating the ratio of total LSR/ β-TUB, total LSR expression in young males appeared to be the highest in the OB (1.22 ± 0.24, Fig 3C), RET (0.908 ± 0.24, Fig 3D), and HT (0.907 ± 0.097, Fig 3A). LSR expression was lower in the CX (0.658 ± 0.176, Fig 3E), HIP (0.581 ± 0.209; Fig 3B), and CB (0.488 ± 0.037; Fig 3F) as compared to the other mentioned regions when compared to their respective β-TUB expression. With aging, no major changes in LSR expression was observed, except in HT and OB were LSR expression was lower by 36.25% (n = 3, *p* = 0.027, Fig 3A) and 30.31% (n = 3, *p* = 0.05, Fig 3C), respectively. However, there was also a tendency of decreased LSR expression by 80.03% in HIP (n = 3, *p* = 0.093, Fig 3B) of 18-month-old male mice, although this did not reach statistical significance.

### LSR expression in neurons and glial cells

**Fig 4:**
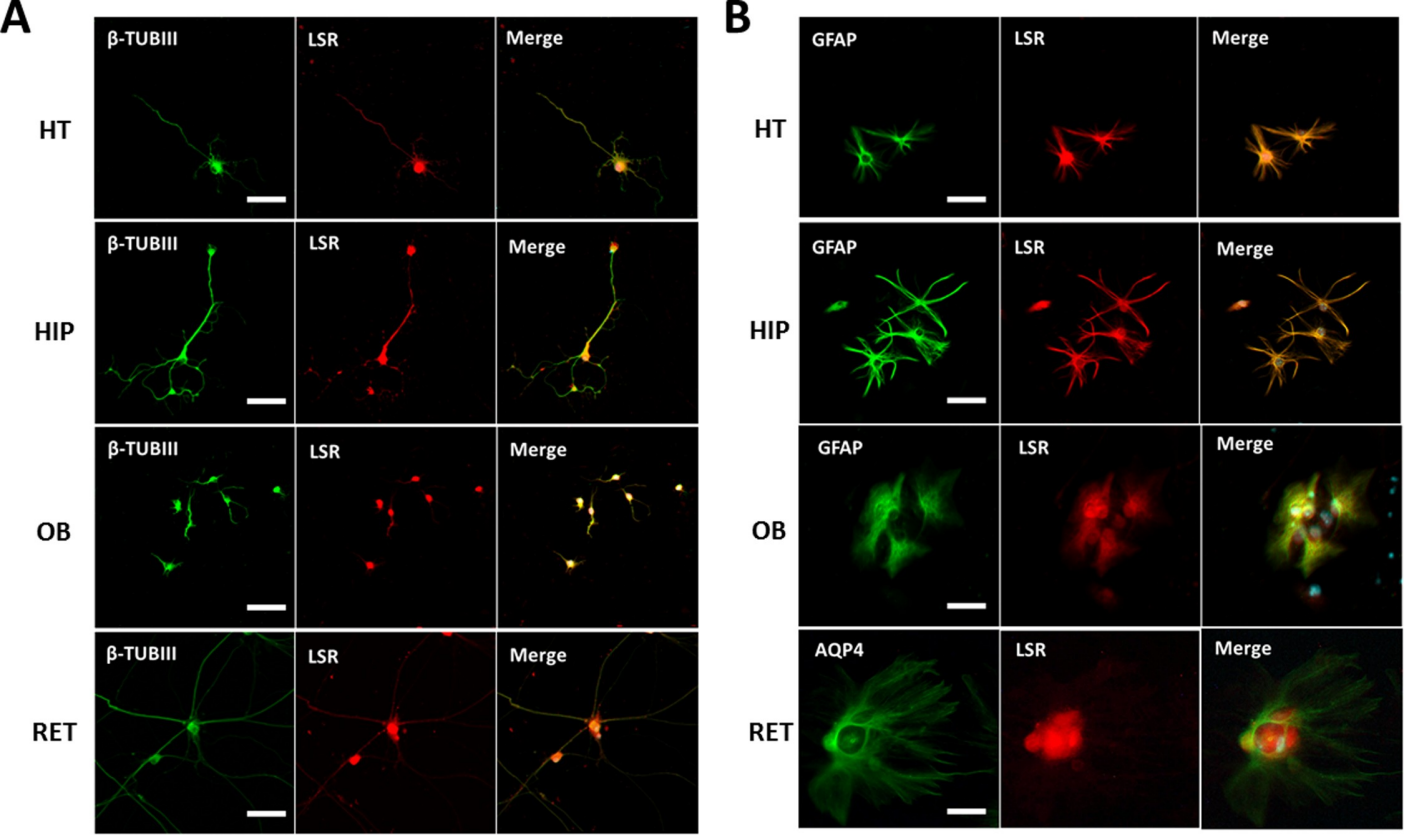
**Immunocytostaining of neuronal (A) and glial (B) cell cultures of different brain regions: HT, HIP, OB, and RET.** Anti-β-III tubulin (β-TUB III) was the primary antibody used to stain neurons, anti-GFAP and anti-AQP4 were used to label astrocytes, and anti-LSR Sigma was used to identify LSR protein. Images were taken using Fvi10 confocal microscope at a 40-X magnification (bar scale = 20 μm).

In view of these results, we performed immunocytostaining of LSR in pure neuronal vs mixed cultures from other CNS regions (Fig 4A). LSR was detected in neurons isolated from HIP, HT, OB, and RET (Fig 4A). LSR staining was observed primarily around cell soma, but some neurites were also weakly stained. On the other hand, immunocytostaining of glial cells from these same regions revealed colocalization of LSR with the glial cell marker, GFAP, as well as the astrocyte marker AQP4 (Fig 4B).

### Glial cells highly express LSR in cerebellum

**Fig 5:**
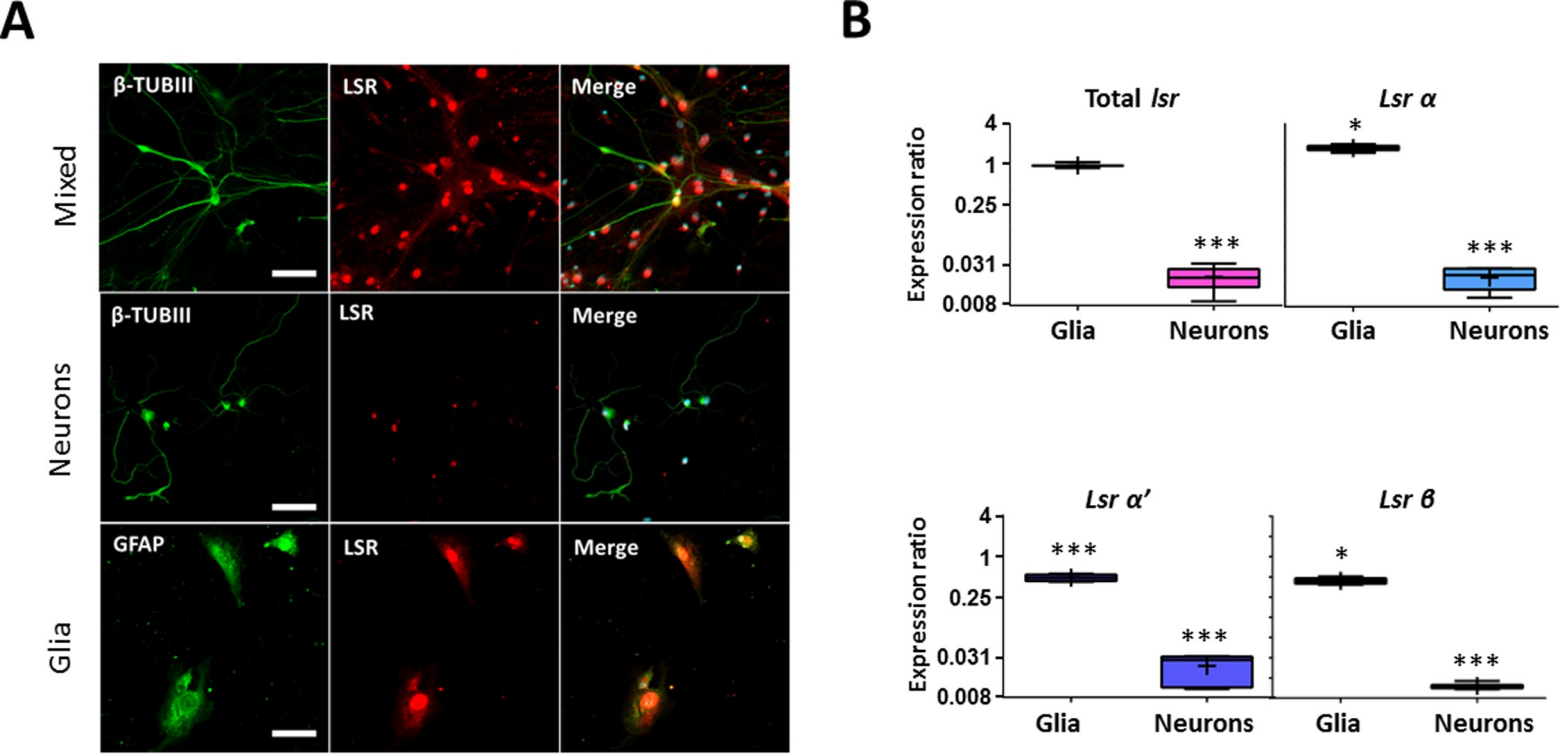
LSR is mainly expressed in glial cells in the CB. (A) Immunocytostaining of primary mixed, pure neuronal and glial cerebellar cell cultures using anti- β-III tubulin (β-TUB III) for neurons, anti-GFAP for astrocytes, and anti-LSR X-25 for LSR. Images were taken using Fvi10 confocal microscope at a 40-X magnification (bar scale = 20 μm). (B) Boxplot representation of expression ratio of total *lsr*, α, α’, and β in cerebellar glial and neuronal cell cultures relative to mixed cerebellar cultures. Statistical significance is represented as: * p ≤ 0.05, ** p≤0.01, *** p ≤ 0.001.

Immunocytostaining of neuronal CB cultures with anti-β-TUB III, a specific neuronal marker and anti-LSR X-25 revealed LSR expression in CB that was relatively weak and mostly located in the soma (Fig 5A). On the other hand, immunocytostaining of glial cell cultures, revealed a strong expression of LSR in this cell type (Fig 5B), with colocalization of GFAP and LSR. RT-qPCR analysis on three different CB cell cultures of neurons and glia were then performed to verify these results (Fig 5B), and confirmed higher *lsr* expression in glia (0.959-fold, *p* = 0.35), as compared to that in neurons (0.02-fold, *p* = 0.0001), when normalized to cerebellar mixed cell cultures. Furthermore, LSR isoforms were differentially expressed in glia, where *lsr α* was upregulated (1.76, *p* = 0.017, Fig 5B), while both *lsr α’* (0.471, *p* = 0.0001, Fig 5B) and β (0.441, *p* = 0.019, Fig 5B) were downregulated relative to cerebellar mixed cultures.

## Conclusions and discussion

Here we demonstrate that LSR expression in the CNS is regio-specific. Each CNS area has its own expression profile for the different LSR chains, thus allowing for specific combination of subunits forming the LSR multimeric complex. Some CNS regions exhibit a stronger LSR expression at the mRNA and/or protein level. Moreover, we demonstrated age-dependent changes in LSR expression, and a strong glia expression of LSR compared to neurons. As previously reported, LSR may play a role in regulation of cholesterol distribution in the CNS [9]. The presence of the BBB prevents access of circulating peripheral lipoproteins to the CNS. Thus, the brain relies on itself to satisfy neuronal needs of cholesterol [5,6]. Adult neurons depend on astrocytes to fulfill those needs [23]. In adult astrocytes, the newly synthesized cholesterol is loaded into ApoE-containing lipoproteins. These HDL-like lipoproteins are needed to export cholesterol from astrocytes, *via* ABCA1 and ABCG1 transporters, to neurons where they bind via ApoE as ligand to lipoprotein receptors, such as LSR, and are internalized through receptor-mediated endocytosis [24]. Here, we show that LSR is differentially expressed across the brain at both RNA and protein levels. At the RNA level, the HT, HIP, OB, and CB all show high levels of total *lsr* RNA expression (Table 1). At the protein level, immunoblots show that the HT, OB, and RET express the highest levels of LSR when normalized to β-TUB, which may reflect a specific need of these regions to tightly regulate cholesterol for proper functioning. It is known that LSR is present in the endothelial cells at tight junctions. Despite the fact that all tissues collected contain blood vessels, the high levels of LSR found in specific brain areas cannot be attributed solely to that found in endothelial cells. Indeed, since these cells are found uniformly throughout the CNS, the differences in LSR expression observed reflect those in CNS cells, and therefore neurons or glial cells. Here, we found that LSR is strongly expressed in glial cells relative to neurons in the CNS, thus suggesting an essential role of this lipoprotein in the cholesterol trafficking between these two cell types. Indeed, while this was clearly demonstrated in the CB, which provided sufficient mRNA to compare *lsr* levels in glial cells and neurons, immunocytostaining of other structures also demonstrated significant LSR protein levels in GFAP-positive cells. In view of this, and based on LSR’s role as lipoprotein receptor, we would hypothesize that the LSR present on glial cells might play a role in the glia-neuron cross talk required for feedback control of cholesterol synthesis, regulation of circulating cholesterol and thus maintenance of proper brain function. Glial LSR may have a possible role in internalizing excess ApoE containing lipoprotein particles excreted from neurons, thus possibly activating a signaling pathway to suppress the synthesis and/or loading of cholesterol onto lipoproteins in glial cells. After internalization, free cholesterol derived from lysosomal processing of ApoE-cholesterol particles [25,26] is transported to membranes. Any excess cholesterol in neurons would then be either uploaded onto ApoE-containing lipoproteins to be exported via ABCG4 to the CSF [24], or esterified into cholesterol esters via acyl-coA cholesterol acyltransferases (ACAT1) to be stored as lipid droplets [27,28], or converted to 24-hydroxycholesterol (24S-OHC) by cholesterol 24-hydroxylase (CYP46A1). Since 24S-OHC can readily cross the BBB, it is transported in the blood to the liver for excretion [29]. An *in vitro* study shows that treatment of activated primary microglial cells with ApoE peptide (EP) caused downregulation of ApoE synthesis in culture [30], which suggests that regulation of cholesterol synthesis and/or transport require a strict mechanism of glial retro-control. If LSR is deficient, such control mechanisms might be perturbed in glia cells leading to upregulation of cholesterol synthesis and increased lipoprotein secretion, leading ultimately to a possible saturation of neurons with cholesterol. Excess of cholesterol might then accumulate as lipid droplets and in neuronal membranes, which could in turn disrupt protein and lipid trafficking required for synapse assembly in neurons causing neurodegeneration [31]. With age, *lsr* RNA expression decreases in both the HT, and HIP. Also, LSR protein levels are clearly downregulated in the HT, and show a tendency to be downregulated in the HIP and OB (Table 2). Those observations are consistent with reported changes in CNS cholesterol during aging and neurodegenerative pathologies. Disturbance of cholesterol homeostasis in the brain is coupled to age-related brain dysfunction including synaptic function, neuronal survival and inflammatory status in the aging brain and may contribute to neurodegenerative pathologies such as Alzheimer disease [32, 33].

**Table 1:** Differential *total lsr* mRNA expression summary.

Target		*VS*	Reference		Region					
Age	Sex	*VS*	Age	Sex	HT	HIP	OB	RET	CX	CB
Y	♂	***VS***	Y Brain	♂	↑	↑	↑	=	= / ↓	↑
O	♂	***VS***	Y	♂	↓	↓	=	=	=	=
Y	♀	***VS***	Y	♂	↓	=		=	↑	=
O	♀	***VS***	O	♂	↑	↑		=	=	=
O	♀	***VS***	Y	♀	↑	= / ↓		= / ↑	=	↓

Summary of *total lsr* mRNA expression in different brain regions of young male versus the whole brain, young versus old male brain, young females versus old males, old females versus old males, and old females versus young females. (Y) young, (O) old, (♂) male, (♀) female, (HT) hypothalamus, (HIP) hippocampus, (OB) olfactory bulb, (RET) retina, (CX) cortex, (CB) cerebellum, (↑) upregulation, (↓) downregulation, (=) no change, (/) tendency, (▄) no data.

**Table 2:** Differential total LSR protein expression in young and old males.

			Region					
Protein	Age	Sex	HT	HIP	OB	RET	CX	CB
**Ratio LSR/β-TUB**	Y	♂	0.91	0.58	1.22	0.91	0.75	0.49
	O	♂	0.57	0.12	0.85	0.83	0.66	0.53

Ratio of LSR (target) over β-TUB (reference). If ratio is equal to 1: equal expression, less than 1: lower, larger than 1: higher. (Y) young, (O) old, (♂) male, (HT) hypothalamus, (HIP) hippocampus, (OB) olfactory bulb, (RET) retina, (CX) cortex, (CB) cerebellum.

Inducible glia-specific and neuron-specific conditional knockout of *lsr* are currently under development to determine if decrease of LSR in the CNS might lead to age-related changes in regiospecific-dependent functions (thermoregulation, sex drive, wake/sleep cycle, or hunger for HT [34]; learning and or memory for HIP [35]; olfactory deficits for OB [36]; and motor control problems for CB [37]).

## Supporting information

S1 Fig Boxplot of RT-qPCR data of *lsr* mRNA expression in different brain regions of 3-month-old female C57Bl/6JRj mice (n = 3) with respect to 3-month-old male C57Bl/6JRj mice (n = 3). (A) Box plot of total *lsr* expression in various regions including hypothalamus (HT), hippocampus (HIP), retina (RET), cortex (CX), and cerebellum (CB). (B) Expression ratio of different *lsr* isoforms α, α’, and β, respectively Statistical significance is represented as: * p ≤ 0.05, ** p ≤ 0.01, *** p ≤ 0.001.
(TIF)

S2 Fig Boxplot of RT-qPCR data of *lsr* mRNA expression in different brain regions of 18-month-old female C57Bl/6JRj mice (n = 3) with respect to 18-month-old male C57Bl/6JRj mice (n = 3). (A) Box plot of total *lsr* expression in various regions including hypothalamus (HT), hippocampus (HIP), retina (RET), cortex (CX), and cerebellum (CB). (B) Expression ratio of different *lsr* isoforms α, α’, and β. Statistical significance is represented as: * p ≤ 0.05, ** p ≤ 0.01, *** p ≤ 0.001.
(TIF)

S3 Fig Boxplot of RT-qPCR data of *lsr* mRNA expression in different brain regions of 18-month-old female C57Bl/6JRj mice (n = 3) with respect to 3-month-old female C57Bl/6JRj mice (n = 3). (A) Box plot of total *lsr* expression in various regions including hypothalamus (HT), hippocampus (HIP), retina (RET), cortex (CX), and cerebellum (CB). (B) Expression ratio of different *lsr* isoforms α, α’, and β. Statistical significance is represented as: * p ≤ 0.05, ** p ≤ 0.01, *** p ≤ 0.001.
(TIF)

S4 Fig Representative immunoblot of LSR expression among different regions of 18-month-old male brain (n = 3). Anti-LSR Sigma antibody was used to detect LSR in different brain regions, including the HT, HIP, OB, Ret, CX, and CB, as indicated. The β-TUB expression of each region is shown below that of LSR.
(TIF)

S1 Table RT-qPCR primers used in the study. Forward and reverse primers ised fpr the three reference genes used *Hprt*, *Pgk1*, and *Tfrc*, and target isoforms of *lsr*, *total* (*T*), *α*, *α’*, and *β*.
(TIF)

## References

[1] Goritz C, Mauch DH, Pfrieger FW. Multiple mechanisms mediate cholesterol-induced synaptogenesis in a CNS neuron. Mol Cell Neurosci. 2005;29: 190–201. 10.1016/j.mcn.2005.02.006 15911344

[2] Fester L, Zhou L, Bütow A, Huber C, von Lossow R, Prange-Kiel J, et al Cholesterol-promoted synaptogenesis requires the conversion of cholesterol to estradiol in the hippocampus. Hippocampus. 2009;19: 692–705. 10.1002/hipo.20548 19156851

[3] de Chaves EI, Rusiñol AE, Vance DE, Campenot RB, Vance JE. Role of lipoproteins in the delivery of lipids to axons during axonal regeneration. J Biol Chem. 1997;272: 30766–30773. 10.1074/jbc.272.49.30766 9388216

[4] Claudepierre T, Pfrieger FW. [New aspects of cholesterol in the central nervous system]. Med Sci MS. 2003;19: 601–605. 10.1051/medsci/2003195601 12836394

[5] Quan G, Xie C, Dietschy JM, Turley SD. Ontogenesis and regulation of cholesterol metabolism in the central nervous system of the mouse. Brain Res Dev Brain Res. 2003;146: 87–98. 14643015 10.1016/j.devbrainres.2003.09.015

[6] Turley SD, Burns DK, Dietschy JM. Preferential utilization of newly synthesized cholesterol for brain growth in neonatal lambs. Am J Physiol. 1998;274: E1099–1105. 10.1152/ajpendo.1998.274.6.E1099 9611162

[7] Fagan AM, Holtzman DM, Munson G, Mathur T, Schneider D, Chang LK, et al Unique lipoproteins secreted by primary astrocytes from wild type, apoE (-/-), and human apoE transgenic mice. J Biol Chem. 1999;274: 30001–30007. 10.1074/jbc.274.42.30001 10514484

[8] Herz J. Apolipoprotein E receptors in the nervous system. Curr Opin Lipidol. 2009;20: 190–196. 10.1097/MOL.0b013e32832d3a10 19433918 PMC2848396

[9] Stenger C, Pinçon A, Hanse M, Royer L, Comte A, Koziel V, et al Brain region-specific immunolocalization of the lipolysis-stimulated lipoprotein receptor (LSR) and altered cholesterol distribution in aged LSR+/- mice. J Neurochem. 2012;123: 467–476. 10.1111/j.1471-4159.2012.07922.x 22909011

[10] Bihain BE, Yen FT. The lipolysis stimulated receptor: a gene at last. Curr Opin Lipidol. 1998;9: 221–224. 9645504 10.1097/00041433-199806000-00006

[11] Yen FT, Masson M, Clossais-Besnard N, André P, Grosset JM, Bougueleret L, et al Molecular cloning of a lipolysis-stimulated remnant receptor expressed in the liver. J Biol Chem. 1999;274: 13390–13398. 10.1074/jbc.274.19.13390 10224102

[12] Yen FT, Roitel O, Bonnard L, Notet V, Pratte D, Stenger C, et al Lipolysis stimulated lipoprotein receptor: a novel molecular link between hyperlipidemia, weight gain, and atherosclerosis in mice. J Biol Chem. 2008;283: 25650–25659. 10.1074/jbc.M801027200 18644789

[13] Narvekar P, Berriel Diaz M, Krones-Herzig A, Hardeland U, Strzoda D, Stöhr S, et al Liver-specific loss of lipolysis-stimulated lipoprotein receptor triggers systemic hyperlipidemia in mice. Diabetes. 2009;58: 1040–1049. 10.2337/db08-1184 19188430 PMC2671043

[14] Mesli S, Javorschi S, Bérard AM, Landry M, Priddle H, Kivlichan D, et al Distribution of the lipolysis stimulated receptor in adult and embryonic murine tissues and lethality of LSR-/- embryos at 12.5 to 14.5 days of gestation. Eur J Biochem. 2004;271: 3103–3114. 10.1111/j.1432-1033.2004.04223.x 15265030

[15] Sohet F, Lin C, Munji RN, Lee SY, Ruderisch N, Soung A, et al LSR/angulin-1 is a tricellular tight junction protein involved in blood-brain barrier formation. J Cell Biol. 2015;208: 703–711. 10.1083/jcb.201410131 25753034 PMC4362448

[16] Martin S, Parton RG. Lipid droplets: a unified view of a dynamic organelle. Nat Rev Mol Cell Biol. 2006;7: 373–378. 10.1038/nrm1912 16550215

[17] Daneman R, Zhou L, Agalliu D, Cahoy JD, Kaushal A, Barres BA. The mouse blood-brain barrier transcriptome: a new resource for understanding the development and function of brain endothelial cells. PloS One. 2010;5: e13741 10.1371/journal.pone.0013741 21060791 PMC2966423

[18] Iwamoto N, Higashi T, Furuse M. Localization of angulin-1/LSR and tricellulin at tricellular contacts of brain and retinal endothelial cells in vivo. Cell Struct Funct. 2014;39: 1–8. 24212375 10.1247/csf.13015

[19] Aranda PS, LaJoie DM, Jorcyk CL. Bleach gel: a simple agarose gel for analyzing RNA quality. Electrophoresis. 2012;33: 366–369. 10.1002/elps.201100335 22222980 PMC3699176

[20] Boda E, Pini A, Hoxha E, Parolisi R, Tempia F. Selection of reference genes for quantitative real-time RT-PCR studies in mouse brain. J Mol Neurosci MN. 2009;37: 238–253. 10.1007/s12031-008-9128-9 18607772

[21] Livak KJ, Schmittgen TD. Analysis of relative gene expression data using real-time quantitative PCR and the 2(-Delta Delta C(T)) Method. Methods San Diego Calif. 2001;25: 402–408. 10.1006/meth.2001.1262 11846609

[22] Steinmetz CC, Buard I, Claudepierre T, Nägler K, Pfrieger FW. Regional variations in the glial influence on synapse development in the mouse CNS. J Physiol. 2006;577: 249–261. 10.1113/jphysiol.2006.117358 16959855 PMC2000689

[23] Zhang J, Liu Q. Cholesterol metabolism and homeostasis in the brain. Protein Cell. 2015;6: 254–264. 10.1007/s13238-014-0131-3 25682154 PMC4383754

[24] Chen J, Zhang X, Kusumo H, Costa LG, Guizzetti M. Cholesterol efflux is differentially regulated in neurons and astrocytes: implications for brain cholesterol homeostasis. Biochim Biophys Acta. 2013;1831: 263–275. 10.1016/j.bbalip.2012.09.007 23010475 PMC3534809

[25] Fagan AM, Holtzman DM. Astrocyte lipoproteins, effects of apoE on neuronal function, and role of apoE in amyloid-beta deposition in vivo. Microsc Res Tech. 2000;50: 297–304. 10.1002/1097-0029(20000815)50:4<297::AID-JEMT9>3.0.CO;2-C 10936884

[26] Ikonen E. Cellular cholesterol trafficking and compartmentalization. Nat Rev Mol Cell Biol. 2008;9: 125–138. 10.1038/nrm2336 18216769

[27] Liu B, Turley SD, Burns DK, Miller AM, Repa JJ, Dietschy JM. Reversal of defective lysosomal transport in NPC disease ameliorates liver dysfunction and neurodegeneration in the npc1-/- mouse. Proc Natl Acad Sci U S A. 2009;106: 2377–2382. 10.1073/pnas.0810895106 19171898 PMC2650164

[28] Bryleva EY, Rogers MA, Chang CCY, Buen F, Harris BT, Rousselet E, et al ACAT1 gene ablation increases 24(S)-hydroxycholesterol content in the brain and ameliorates amyloid pathology in mice with AD. Proc Natl Acad Sci U S A. 2010;107: 3081–3086. 10.1073/pnas.0913828107 20133765 PMC2840286

[29] Meaney S, Bodin K, Diczfalusy U, Björkhem I. On the rate of translocation in vitro and kinetics in vivo of the major oxysterols in human circulation: critical importance of the position of the oxygen function. J Lipid Res. 2002;43: 2130–2135. 10.1194/jlr.m200293-jlr200 12454275

[30] Pocivavsek A, Burns MP, Rebeck GW. Low Density Lipoprotein Receptors Regulate Microglial Inflammation Through C-Jun N-Terminal Kinase. Glia. 2009;57: 444–453. 10.1002/glia.20772 18803301 PMC2628955

[31] Pennetta G, Welte MA. Emerging Links between Lipid Droplets and Motor Neuron Diseases. Dev Cell. 2018;45: 427–432. 10.1016/j.devcel.2018.05.002 29787708 PMC5988256

[32] Ledesma MD, Martin MG, Dotti CG. Lipid changes in the aged brain: effect on synaptic function and neuronal survival. Prog Lipid Res. 2012;51:23–35. 10.1016/j.plipres.2011.11.004 22142854

[33] Martin M, Dotti CG, Ledesma MD. Brain cholesterol in normal and pathological aging. Biochim Biophys Acta. 2010 8;1801(8):934–44. 10.1016/j.bbalip.2010.03.011 20359547

[34] Saper CB, Lowell BB. The hypothalamus. Curr Biol. 2014;24: R1111–R1116. 10.1016/j.cub.2014.10.023 25465326

[35] Leuner B, Gould E. Structural Plasticity and Hippocampal Function. Annu Rev Psychol. 2010;61: 111–C3. 10.1146/annurev.psych.093008.100359 19575621 PMC3012424

[36] Lledo P-M, Merkle FT, Alvarez-Buylla A. Origin and function of olfactory bulb interneuron diversity. Trends Neurosci. 2008;31: 392–400. 10.1016/j.tins.2008.05.006 18603310 PMC4059175

[37] Sullivan EV. Cognitive Functions of the Cerebellum. Neuropsychol Rev. 2010;20: 227–228. 10.1007/s11065-010-9144-8 20811946 PMC4723423

