# Supplementary figures and images for: Age-related changes in regiospecific expression of Lipolysis Stimulated Receptor (LSR) in mice brain
Source: PLoS One. 2019 Jun 24;14(6):e0218812. doi: 10.1371/journal.pone.0218812 (PMC6590887; doi:10.1371/journal.pone.0218812)

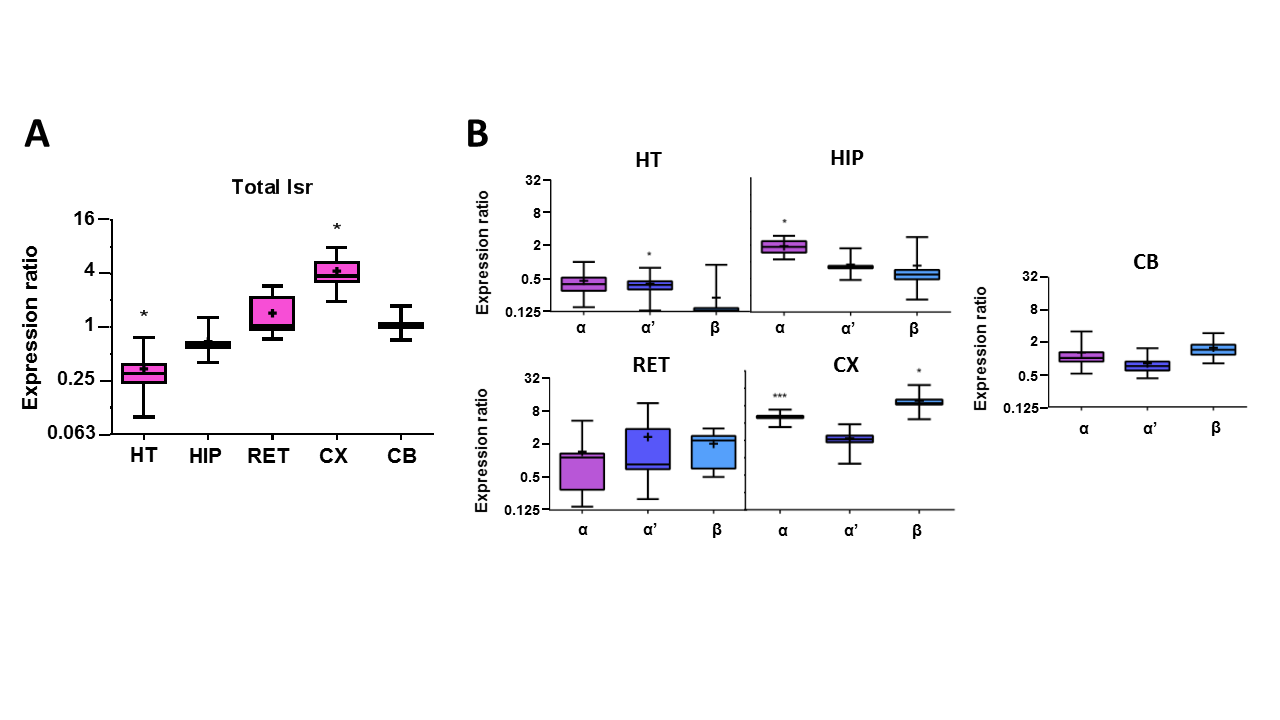

Supplement: S1 Fig — (A) Box plot of total lsr expression in various regions including hypothalamus (HT), hippocampus (HIP), retina (RET), cortex (CX), and cerebellum (CB). (B) Expression ratio of different lsr isoforms α, α’, and β, respectively Statistical significance is represented as: * p ≤ 0.05, ** p ≤ 0.01, *** p ≤ 0.001. (TIF) [file pone.0218812.s001.tif]

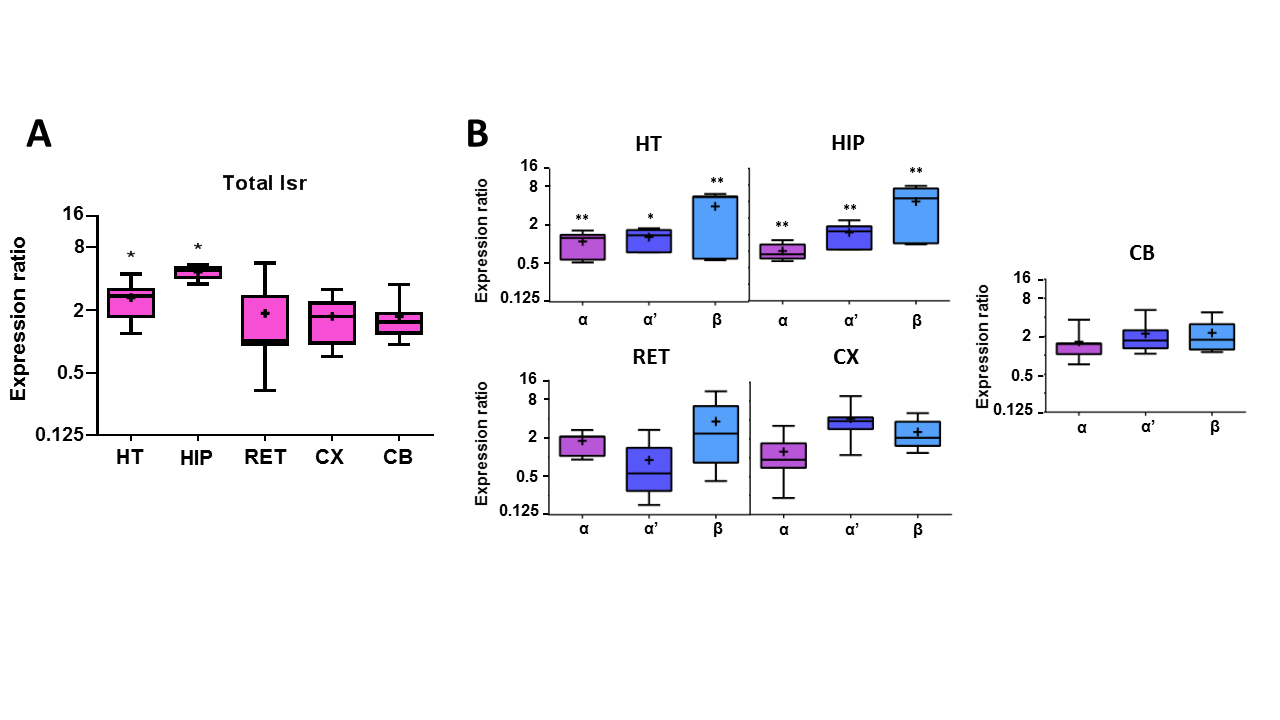

Supplement: S2 Fig — (A) Box plot of total lsr expression in various regions including hypothalamus (HT), hippocampus (HIP), retina (RET), cortex (CX), and cerebellum (CB). (B) Expression ratio of different lsr isoforms α, α’, and β. Statistical significance is represented as: * p ≤ 0.05, ** p ≤ 0.01, *** p ≤ 0.001. (TIF) [file pone.0218812.s002.tif]

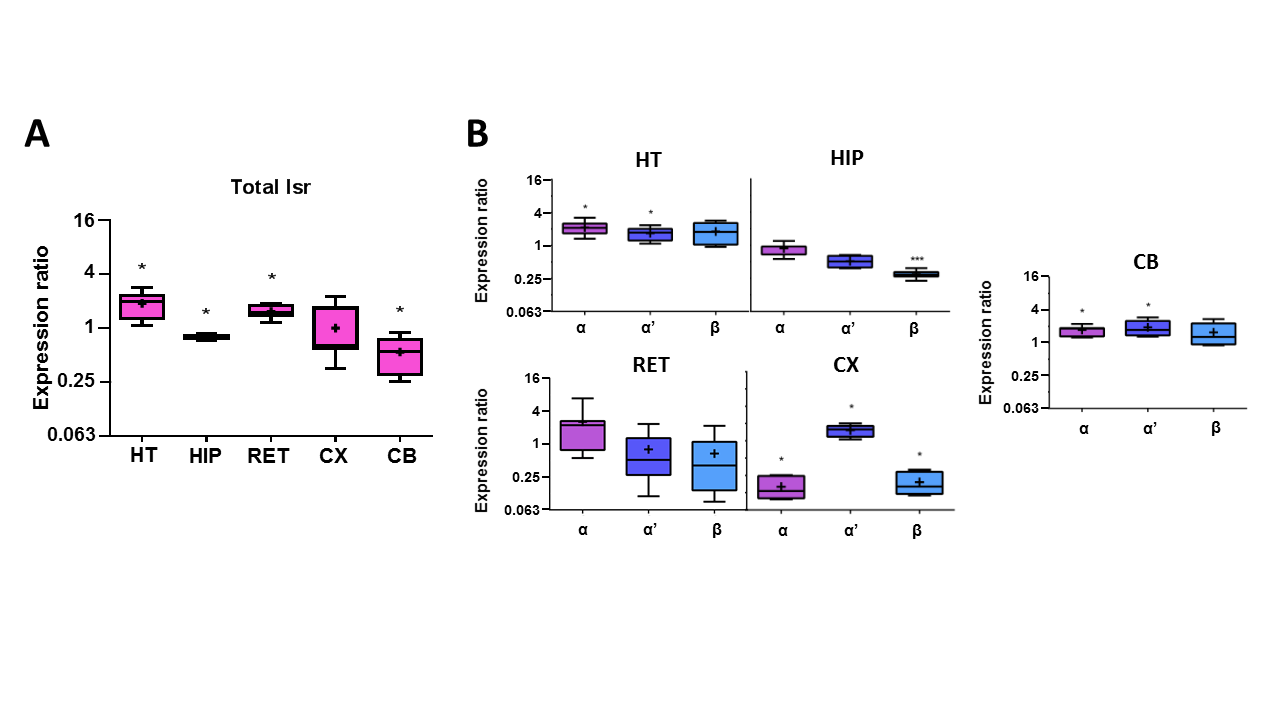

Supplement: S3 Fig — (A) Box plot of total lsr expression in various regions including hypothalamus (HT), hippocampus (HIP), retina (RET), cortex (CX), and cerebellum (CB). (B) Expression ratio of different lsr isoforms α, α’, and β. Statistical significance is represented as: * p ≤ 0.05, ** p ≤ 0.01, *** p ≤ 0.001. (TIF) [file pone.0218812.s003.tif]

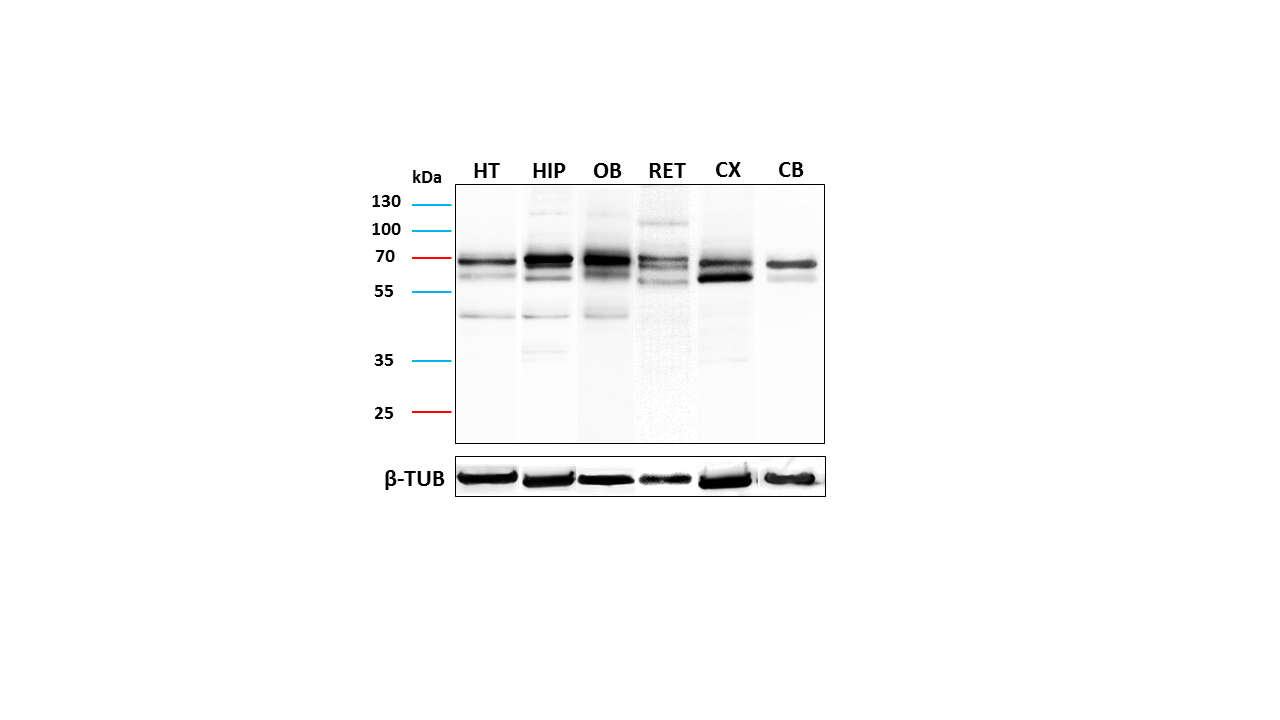

Supplement: S4 Fig — Anti-LSR Sigma antibody was used to detect LSR in different brain regions, including the HT, HIP, OB, Ret, CX, and CB, as indicated. The β-TUB expression of each region is shown below that of LSR. (TIF) [file pone.0218812.s004.tif]

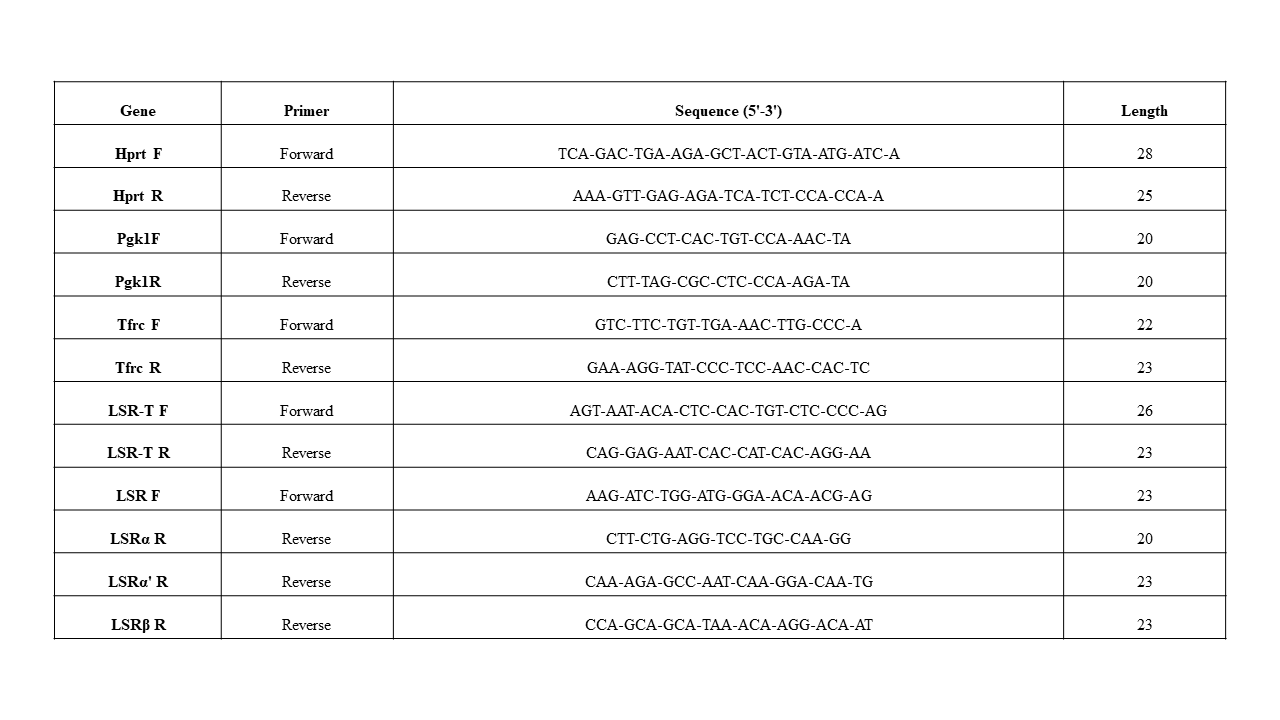

Supplement: S1 Table — Forward and reverse primers ised fpr the three reference genes used Hprt, Pgk1, and Tfrc, and target isoforms of lsr, total (T), α, α’, and β. (TIF) [file pone.0218812.s005.tif]
